# Predictors of Five-Year Outcomes in Patients with Acute Coronary Syndromes

**DOI:** 10.3390/jcdd12060234

**Published:** 2025-06-18

**Authors:** Luca Di Vito, Giancarla Scalone, Federico Di Giusto, Filippo Bruscoli, Simona Silenzi, Adelina Selimi, Arianna Massari, Domenico Delfino, Federico Guerra, Pierfrancesco Grossi

**Affiliations:** 1 Cardiology Unit, C. and G. Mazzoni Hospital, 63100 AST Ascoli Piceno, Italy; 2 Cardiology and Arrhythmology Clinic, Marche Polytechnic University of Ancona, 60121 Ancona, Italy

**Keywords:** residual risk, sex-related differences, acute coronary syndrome, diabetes mellitus, chronic kidney disease

## Abstract

Background: Residual risk after acute coronary syndromes (ACSs) continues to affect prognosis. We investigated the impact of female sex, non-ST-segment–elevation myocardial infarction (NSTEMI), diabetes mellitus (DM), and chronic kidney disease (CKD) on coronary atherosclerosis extent, culprit stenosis location, and bio-humoral data. The rate of both major adverse cardiovascular events (MACE) and non-fatal recurrent coronary events (RCE) was additionally evaluated. Methods: We enrolled 1404 ACS patients and followed them for up to 5 years. Coronary culprit and non-culprit stenoses were analyzed using angiography. Biohumoral data was assessed at admission and at 1 month and 12 months after discharge. Patients were compared based on sex, NSTEMI, DM, and CKD presence. Results: NSTEMI patients had a higher number of total coronary stenoses (3.5 vs. 3.3, *p* = 0.013) and non-culprit stenoses (2.3 vs. 1.6, *p* = 0.0001). Non-culprit percent stenosis was significantly greater in NSTEMI as compared to STEMI patients (57.9% vs. 47.1%, *p* = 0.0001). DM patients had a higher frequency of bifurcation lesions (41% vs. 25%, *p* = 0.0001). CKD patients showed a higher prevalence of left main disease (3.4% vs. 1.5%, *p* = 0.038). Female patients had higher LDL-cholesterol values at 1 month and 12 months. NSTEMI, DM, and creatinine level were independent predictors of MACE. NSTEMI patients had an increased risk of non-fatal RCE. Conclusions: NSTEMI, DM, and creatinine levels at admission were independent predictors of MACE in the first 5 years after an ACS.

## 1. Introduction

The term “residual risk” following acute coronary syndromes (ACSs) denotes the incidence of cardiovascular events in patients receiving lipid-lowering therapies [1], often attributed to inadequate blood pressure or plasma lipid control, subclinical inflammation, uncontrolled diabetes mellitus (DM), and environmental factors [1].

As a result of improvements in ACS treatment and post-hospital discharge management, the risk of recurrent coronary events has been reduced. Our group previously demonstrated that in a contemporary cohort of ST-segment–elevation myocardial infarction (STEMI) patients, the incidence of non-fatal recurrent coronary events (RCE) reached 7.6% at the 5-year follow-up time [2].

Although many studies have explored prognosis after ACS [3,4,5,6,7,8,9,10], key knowledge gaps remain. These include the long-term outcomes in underrepresented populations (e.g., women) [11], the effectiveness of therapies in specific subgroups (e.g., statin therapy in diabetic or chronic kidney disease (CKD) patients) [12], and the independent contribution of each residual risk factor, as these factors often cluster together [13,14]. We aimed to assess the distinct impact of female sex, non-ST-segment-elevation myocardial infarction (NSTEMI), DM, and CKD on clinical, angiographic, and biochemical features, as well as their influence on 5-year outcomes.

## 2. Materials and Methods

### 2.1. Study Population

At C. and G. Mazzoni Hospital, AST Ascoli Piceno, 1404 patients who presented with their first ACS and underwent percutaneous coronary intervention (PCI) between January 2015 and December 2018 were retrospectively included.

The American Heart Association/American College of Cardiology guidelines classified ACSs as either NSTEMI or STEMI. Non–ST-segment–elevation ACS or unstable angina were the two criteria used to identify NSTEMI [15,16].

For the treatment of native and de novo coronary stenoses, all patients underwent PCI using second-generation drug-eluting stents.

Patients with a history of coronary artery bypass graft and those with significant restenosis or thrombosis of a previous stent as the culprit lesion at admission were excluded based on patient-related criteria. The distribution of cardiovascular risk factors and biometric data at hospital admission was documented.

Medications taken 12 months after hospital discharge were collected.

Informed consent for research participation was obtained before coronary angiography and from identifiable subjects.

The research was carried out in accordance with the World Medical Association Declaration of Helsinki, and the study was authorized by the Marche region’s ethical committee in Italy (permission number 2020032023).

### 2.2. Residual Risk and Clinical Endpoints

The entire studied population was analyzed based on four determinants of the residual risk: sex-related differences (male vs. female patients), clinical presentation (STEMI vs. NSTEMI), type 2 DM presence, and CKD presence.

In this study, the term “sex” was defined following the Sex and Gender Equity in Research.

(SAGER) guidelines [17], and it refers to its documentation in the patient’s medical file. Sex is recorded as either female or male.

A patient was diagnosed with DM if at least one of the following requirements was satisfied: a documented history of the disease, the use of hypoglycemia medications, a fasting glucose level greater than 126 mg/dL, hemoglobin A1c of 6.5%, a 2 h plasma glucose level greater than 200 mg/dL on the oral glucose tolerance test, or classic symptoms with a casual plasma glucose level greater than 200 mg/dL [18].

The Kidney Disease Improving Global Outcomes (KDIGO) criteria and stages were used to diagnose CKD, which includes [19] (1) kidney damage indicators as an albumin/creatinine ratio ≥ 30 mg/g, or (2) estimated glomerular filtration rate (eGFR)  <  60 mL/min/1.73 m^2^.

We estimated eGFR using the modification of diet in renal disease (MDRD) formula [20].

The main clinical outcome of the study was the rate of MACE, which included death from all causes and non-fatal RCE.

Any subsequent hospitalization for acute or chronic coronary syndrome that necessitated coronary angiography or unscheduled PCI was considered a non-fatal RCE. The two secondary clinical endpoints were the individual components of the composite outcome (non-fatal RCE and death from any cause). Clinical follow-up was acquired by clinical visits, telephone calls, hospital databases, or administrative regional data.

### 2.3. Coronary Angiographic Analysis

A coronary stenosis was defined as a distinctive luminal narrowing, determining a percent diameter stenosis (DS) greater than 30%. The number of coronary stenoses in both culprit and non-culprit vessels was assessed on a per-segment basis and reported as the total number of coronary stenoses, as well as the total number of culprit and non-culprit stenoses. The culprit lesion was identified by electrocardiographic ST-segment changes, regional wall motion abnormalities on echocardiographic exam, and/or angiography.

Quantitative coronary angiography (QCA) was conducted offline on a single, end-diastolic 2D imaging frame, analyzed with validated software (QCA-CMS, MEDIS medical imaging systems, Versione 5.1) utilizing an automated edge-detection algorithm. The analysis was independently performed by two experienced observers (L.D.V and F.D.G.) who were blinded to patient groups.

The diameters of the vessel were computed as absolute values (mm). The vessel contours were automatically obtained, and manual adjustment was used if the automated analysis was inaccurate. The computed-estimated initial arterial dimensions at the stenosis site served as the basis for the reference vessel diameter. The following angiographic parameters were obtained for the culprit stenosis: reference vessel diameter (RVD), which was determined by averaging the proximal and distal values; minimum lumen diameter (MLD); percent lesion diameter stenosis; and lesion length.

For the non-culprit stenoses, the percentage of lesion diameter stenosis was obtained.

A culprit stenosis involving a bifurcation was described if there was no lesion-free space between the main branch and the side branch ostium [21].

Culprit stenosis location was categorized as left main (LM) coronary artery, left anterior descending artery, left circumflex artery, and right coronary artery.

Information on the number of stents and stent diameter deployed at the time of the culprit stenosis PCI was collected.

### 2.4. Biohumoral Data

Three blood tests were performed on each patient at three time points: during their hospital stay, 1 month later, and 12 months after hospital discharge.

Triglycerides, LDL-cholesterol, HDL-cholesterol, creatinine, glycemia, hemoglobin A1c, hemoglobin, white blood cell count, fibrinogen, platelets, and uric acid were among the routine blood tests obtained on admission to the hospital.

C-reactive protein and peak high-sensitivity troponin I were also evaluated between 48 and 72 h after admission.

Routine laboratory tests were performed at 1-month and 12-month follow-up periods to measure triglycerides, LDL-cholesterol, HDL-cholesterol, creatinine, glycemia, hemoglobin A1c, and hemoglobin.

### 2.5. Statistical Analysis

SPSS 21.0 was used for statistical analysis (SPSS, Inc., Chicago, IL, USA).

The Chi-square test was used to examine categorical variables, which were represented as absolute and relative frequencies. The Kolmogorov–Smirnov test for normality was conducted to determine whether the continuous variables were normally distributed; as a result, they were given as mean and standard deviation (SD), which were then compared using the *t*-test.

To evaluate the effect of both time and residual risk determinants on LDL-cholesterol levels over the three time periods (hospitalization, one-month, and twelve-month follow-up times), a mixed between-within-subject analysis of variance (ANOVA) was performed.

To evaluate the effect of a set of parameters on MACE (dependent variable), a multivariate logistic regression was used. First, a univariate analysis was conducted, and any variable that showed a *p* < 0.10 was entered en bloc in the multivariate model, along with age as a background variable.

The chi-square test was used to evaluate the model’s ability to differentiate between patients with MACE and those without. The amount of variation in the dependent variable that the model explained was determined using the Cox and Snell R square and Nagelkerke R square.

Survival curves of MACEs for subjects with or without the investigated residual risk determinants were generated using the Kaplan–Meier method, and comparisons were made by applying the log-rank test.

A *p*-value of less than 0.05 (two-tailed) was considered significant.

## 3. Results

### 3.1. Clinical and Angiographic Features Associated with the Four Residual Risk Determinants

Table 1 shows the clinical features, and Table 2 reports angiographic data for the entire studied population.

#### 3.1.1. Sex-Related Differences

Female patients were significantly older (73.9 years vs. 66.9 years, *p* = 0.0001) and had a higher frequency of CKD (43.6% vs. 27.8%, *p* = 0.0001) and DM (29.5% vs. 22.8%, *p* = 0.015) as compared to male patients. Female patients were on insulin more frequently (11.5% vs. 7.9%, *p* = 0.049). On the contrary, female patients were more commonly non-smokers (67.9% vs. 59.6%, *p* = 0.011).

The culprit RVD was significantly lower in female patients (3.1 mm vs. 3.2 mm, *p* = 0.002), as for stent diameter (3.1mm vs. 3.2 mm, *p* = 0.0001), as compared to male patients.

#### 3.1.2. Clinical Presentation

NSTEMI patients were significantly older (70.6 years vs. 67.1 years, *p* = 0.0001) and had a higher frequency of CKD (38.5% vs. 26.8%, *p* = 0.0001) as compared to STEMI patients. Additionally, they were more frequently non-smokers (68.3% vs. 56.9%, *p* = 0.0001), and dyslipidemia was less common (42.6% vs. 52.2%, *p* = 0.0001). NSTEMI patients were less frequently on ticagrelor (15.4% vs. 31.5%, *p* = 0.0001), but they were more commonly treated with insulin (11.4% vs. 6.9%, *p* = 0.004).

NSTEMI patients had a higher number of total coronary stenoses (3.5 stenoses vs. 3.3 stenoses, *p* = 0.013) and non-culprit stenoses (2.3 stenoses vs. 1.6 stenoses, *p* = 0.0001). Culprit lesion stenosis, MLD, and lesion length were significantly lower in NSTEMI patients, while LM involvement (3.1% vs. 1.3%, *p* = 0.0001) as well as bifurcation lesions (37% vs. 23%, *p* = 0.0001) were significantly higher in NSTEMI patients. Non-culprit lesion stenosis was significantly greater in NSTEMI as compared to STEMI patients (57.9% vs. 47.1%, *p* = 0.0001). The total number of implanted stents was higher in NSTEMI patients (1.4 stents vs. 1.2 stents, *p* = 0.0001).

#### 3.1.3. Diabetes Mellitus Presence

Diabetic patients were older (69.8 years vs. 68.2 years, *p* = 0.039), and had a higher BMI (27.7 Kg/m^2^ vs. 27.1 Kg/m^2^, *p* = 0.02) as compared to non-diabetic patients. They were less frequently on ticagrelor (13.4% vs. 28.5%, *p* = 0.001) and were less commonly male subjects (70.1% vs. 76.8%, *p* = 0.015).

DM patients more frequently had a bifurcation lesion as the culprit stenosis (41% vs. 25%, *p* = 0.0001).

#### 3.1.4. Chronic Kidney Disease Presence

CKD patients were significantly older (72.9 years vs. 66.6 years, *p* = 0.0001) and showed a higher frequency of NSTEMI presentation (50.9% vs. 37.7%, *p* = 0.0001) as compared to non-CKD patients. They were more commonly non-smokers (72.4% vs. 56.6%, *p* = 0.0001). They were more commonly on ezetimibe (31.4% vs. 24.3%, *p* = 0.006) and on insulin (17.9% vs. 4.5%, *p* = 0.0001). CKD patients were less likely to be male (65.9% vs. 79.5%, *p* = 0.0001). CKD patients showed a higher LM involvement (3.4% vs. 1.5%, *p* = 0.038).

### 3.2. Laboratory Results Associated with the 4 Residual Risk Determinants

Table 3 displays the laboratory data collected at hospital admission, and Table 4 shows the laboratory results obtained at 1-month and 12-month follow-up periods.

#### 3.2.1. Sex-Related Differences

Female patients had a higher HDL value (47 mg/dL vs. 42.3 mg/dL, *p* = 0.0001) at admission, as was the case for glycemia (127 mg/dL vs. 102 mg/dL, *p* = 0.0001), hemoglobin A1c (6.5% vs. 6.2%, *p* = 0.004), and platelets (248 × 10^3^/µL vs. 220 × 10^3^/µL, *p* = 0.0001) as compared to male patients. Conversely, female patients had a lower value of hemoglobin (12.5 g/dL vs. 14.2 g/dL, *p* = 0.0001), and uric acid (5.8 mg/dL vs. 6.2 mg/dL, *p* = 0.001).

At 1-month and 12-month follow-up periods, female patients showed a significantly higher value of LDL-cholesterol (85 mg/dL vs. 77 mg/dL, *p* = 0.0001 and 82 mg/dL vs. 74 mg/dL, *p* = 0.006, respectively), HDL (48 mg/dL vs. 42 mg/dL, *p* = 0.0001 and 49 mg/dL vs. 43 mg/dL, *p* = 0.001, respectively), and platelets (259 × 10^3^/µL vs. 215 × 10^3^/µL, *p* = 0.0001 and 252 × 10^3^/µL vs. 214 × 10^3^/µL, *p* = 0.001, respectively), while hemoglobin (12 g/dL vs. 14 g/dL, *p* = 0.0001 and 12 g/dL vs. 14 g/dL, *p* = 0.001, respectively) was lower as compared to male patients.

#### 3.2.2. Clinical Presentation

NSTEMI patients had a higher mean value of triglycerides (140 mg/dL vs. 126 mg/dL, *p* = 0.001) as well as glycemia (115 mg/dL vs. 96 mg/dL, *p* = 0.0001), hemoglobin A1c (6.5% vs. 6.1%, *p* = 0.017), and uric acid (6.2 mg/dL vs. 6.0 mg/dL, *p* = 0.026) as compared to STEMI patients at admission.

On the contrary, NSTEMI patients had a lower mean value of LDL-cholesterol (121 mg/dL vs. 126 mg/dL, *p* = 0.015), platelets (219 × 10^3^/µL vs. 233 × 10^3^/µL, *p* = 0.0001), hemoglobin (13.6 g/dL vs. 13.9 g/dL, *p* = 0.0001), white blood cell count (8.7 × 1000/mm^3^ vs. 11.2 × 1000/mm^3^, *p* = 0.0001), C-reactive protein (2.1 mg/dL vs. 4.5 mg/dL, *p* = 0.0001), fibrinogen (429 mg/dL vs. 543 mg/dL, *p* = 0.0001), and peak troponin I (705 ng/L vs. 3500 ng/L, *p* = 0.0001).

At 1-month and 12-month follow-up periods, NSTEMI patients showed significantly higher creatinine values (1.2 mg/dL vs. 0.9 mg/dL, *p* = 0.0001 and 1.4 mg/dL vs. 1.0 mg/dL, *p* = 0.015, respectively) and glycemia (116 mg/dL vs. 107 mg/dL, *p* = 0.0001 and 119 mg/dL vs. 109 mg/dL, *p* = 0.002, respectively), while hemoglobin values were lower (13.4 g/dL vs. 13.9 g/dL, *p* = 0.0001 and 13.4 g/dL vs. 14.1 g/dL, *p* = 0.001, respectively) as compared to STEMI patients.

#### 3.2.3. Diabetes Mellitus Presence

DM patients had a higher mean value of triglycerides (145 mg/dL vs. 128 mg/dL, *p* = 0.003) as well as uric acid (6.3 mg/dL vs. 6.0 mg/dL, *p* = 0.047) as compared to non-diabetic patients at admission.

Conversely, DM patients had a lower mean value of LDL-cholesterol (118 mg/dL vs. 126 mg/dL, *p* = 0.003) and hemoglobin (13.4 g/dL vs. 13.9 g/dL, *p* = 0.0001).

At 1-month and 12-month follow-up periods, DM patients showed significantly lower hemoglobin values (13.3 g/dL vs. 13.8 g/dL, *p* = 0.0001 and 13.3 g/dL vs. 13.8 g/dL, *p* = 0.007, respectively) as compared to non-diabetic patients.

#### 3.2.4. Chronic Kidney Disease Presence

CKD patients had a higher mean value of glycemia (113 mg/dL vs. 105 mg/dL, *p* = 0.041) as well as uric acid (6.3 mg/dL vs. 6.0 mg/dL, *p* = 0.007) compared to non-CKD patients at admission.

Conversely, CKD patients had a lower mean value of LDL-cholesterol (116 mg/dL vs. 127 mg/dL, *p* = 0.001) and hemoglobin (13.1 g/dL vs. 14.1 g/dL, *p* = 0.0001).

At 1-month and 12-month follow-up periods, CKD patients showed significantly higher glycemia values (117 mg/dL vs. 109 mg/dL, *p* = 0.022 and 123 mg/dL vs. 111 mg/dL, *p* = 0.006), while they had lower hemoglobin values (13.0 g/dL vs. 14.0 g/dL, *p* = 0.0001 and 12.9 g/dL vs. 14.0 g/dL, *p* = 0.001, respectively) as compared to non-diabetic patients.

#### 3.2.5. LDL-Cholesterol Reduction in the 4 Residual Risk Determinant Groups

**Figure 1:**
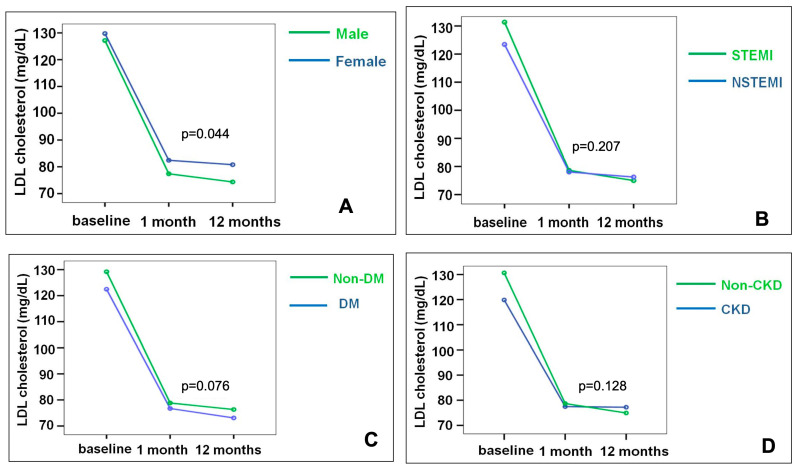
Mean LDL-cholesterol values at hospital admission, 1-month, and 12-month follow-up according to the four residual risk determinants. Panel (**A**): Male vs. Female (main effect *p* = 0.044). Panel (**B**): STEMI vs. NSTEMI (main effect *p* = 0.207). Panel (**C**): non-DM vs. DM (main effect *p* = 0.076). Panel (**D**): Non-CKD vs. CKD (main effect *p* = 0.128). Time effect for all groups: *p* < 0.0001 (Wilk’s Lamda = 0.466). Mixed between-within ANOVA was used.

The mixed between-within ANOVA showed a significant impact of time on LDL-cholesterol reduction in both male and female patients (Wilks’ Lamda for time was 0.466, *p* = 0.0001). The main effect of sex on LDL-cholesterol reduction was statistically significant, resulting in a lower LDL-cholesterol decrease in female patients over time (F = 3.7, *p* = 0.044) (Figure 1).

NSTEMI, DM, and CKD on LDL-cholesterol showed a significant reduction over time (*p* = 0.0001, *p* = 0.0001, *p* = 0.0001), while their main effects on LDL-cholesterol reduction were not statistically significant (*p* = 0.207, *p* = 0.076, and *p* = 0.128, respectively).

### 3.3. MACE Association with the Determinants of the Residual Risk

Table 5 reports the rate of primary and secondary clinical endpoints based on the four determinants of the residual risk. Mean follow-up duration was 61.3 months ±13.6 for the entire studied population. MACE was observed in 485 (34.5%) patients out of 1404 studied patients.

#### 3.3.1. Sex-Related Differences

Female patients had a significantly higher rate of MACE as compared to male patients (43% vs. 31.7%, *p* = 0.0001). Death from any cause was significantly more frequent in female patients (34.1% vs. 24%, *p* = 0.001).

#### 3.3.2. Clinical Presentation

NSTEMI patients had a significantly higher rate of MACE as compared to STEMI patients (43.5% vs. 31.7%, *p* = 0.0001). Both death from any cause and non-fatal RCE were significantly more frequent in NSTEMI patients (31.2% vs. 23%, *p* = 0.001 and 17.3% vs. 6.1%, *p* = 0.0001).

#### 3.3.3. Diabetes Mellitus Presence

DM patients had a significantly higher rate of MACE as compared to non-DM patients (40.7% vs. 32.5%, *p* = 0.006). A non-significant trend for a higher rate of non-fatal RCE was observed in DM patients.

#### 3.3.4. Chronic Kidney Disease Presence

CKD patients had a significantly higher rate of MACE as compared to non-CKD patients (47.1% vs. 28.7%, *p* = 0.0001). Death from any cause was significantly more frequent in CKD patients (41.5% vs. 19.5%, *p* = 0.0001).

### 3.4. Predictors of MACE

Table 6 shows the results of both univariate and multivariate logistic regression with MACE as the dependent variable.

**Figure 2:**
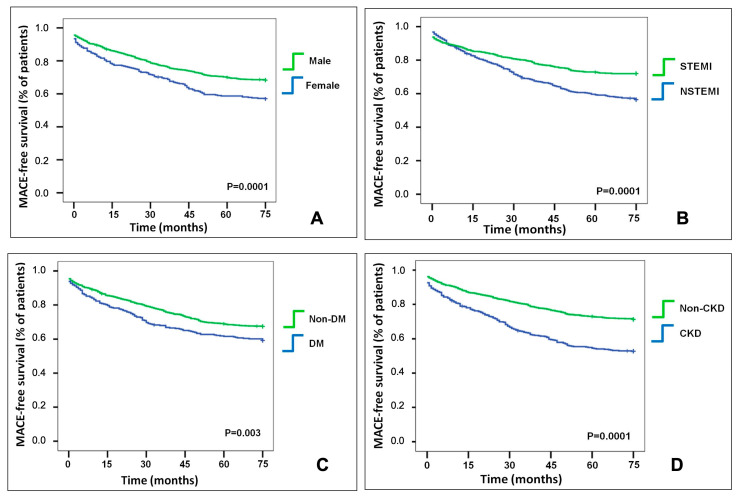
Kaplan–Meier curves for MACEs based on residual risk determinants. Panel (**A**): Female vs. Male (log-rank *p* = 0.0001). Panel (**B**): NSTEMI vs. STEMI (log-rank *p* = 0.0001). Panel (**C**): DM vs. Non-DM (log-rank *p* = 0.006). Panel (**D**): CKD vs. non-CKD (log-rank *p* = 0.0001). Comparisons were performed using the log-rank test.

Comparisons of the Kaplan–Meier curves showed that patients presenting with any of the four residual risk determinants had lower MACE-free survival compared to those without (Figure 2).

A multivariate analysis was conducted to identify independent predictors of MACE. NSTEMI presentation (compared to STEMI), DM, female sex, CKD, age, weight, height, total number of coronary stenoses, number of culprit stenoses, stent diameter, non-culprit lesion stenosis, number of non-culprit stenoses, LDL-cholesterol at admission, HDL at admission, glycemia at admission, hemoglobin at admission, creatinine at admission, and peak high-sensitivity troponin I were among the 18 variables that were included in the final multivariate model. The chi-square value of 22 (8), *p* = 0.005, validated the statistical significance of the entire model with all predictors. The strongest independent predictor of MACE was NSTEMI as a clinical presentation (O.R. = 2.48), followed by DM presence (O.R. = 2.44), low hemoglobin level (O.R. = 0.89), and high creatinine values (O.R. = 1.54). Female sex was not an independent predictor (Table 6).

## 4. Discussion

The present study identified two main results:

1. The four residual risk determinants (female sex, NSTEMI, DM, and CKD) clustered and were associated with advanced age.
2. NSTEMI, DM, and creatinine levels were independent predictors of MACE, while female sex was not. A lower hemoglobin level at admission was an independent predictor of MACE.

### 4.1. Clustering of Risk Factors and Sex Differences in LDL-Cholesterol Reduction and MACEs

A previous study reported a cumulative risk of MACE in a cohort of ACS patients based on the number of comorbidities. Patients with at least three comorbidities have double the risk as compared to patients without comorbidities [3]. We showed a clustering effect of the investigated residual risk determinants.

In the present study, female sex was not an independent predictor for MACE after correcting for comorbidities.

Historically, female patients were considered at a higher risk of MACE after ACSs due to reduced access to both invasive strategies and guideline-driven medical treatments as compared to male patients [22,23]. This undertreatment can be explained by the increased age of ACS presentation and the greater burden of comorbidities typically seen in female patients [22,23].

In our study, all patients underwent PCI with second-generation stents and were discharged on potent P2Y12 inhibitors and on high-intensity lipid-lowering drugs. As a result, there was not a sex-based difference in both invasive and medical treatments. Recently, the Scottish National Data-Linkage Study showed in a large ACS population that women had more chronic conditions and were older than men. There were no sex differences in in-hospital mortality after adjusting for other variables. At a long-term follow-up time, women were 18% less likely than males to present with cardiovascular death and 8% less likely to die from any cause [14].

We also observed that although female and male patients had similar hospital admission values of LDL-cholesterol, female patients showed higher values of LDL-cholesterol at the 1- and 12-month follow-up times. Previous works reported a lower LDL-cholesterol reduction in female patients as compared to male patients after adjusting for lipid-lowering therapy intensity [24,25]. Different causes have been reported, such as a higher rate of statin discontinuation due to side effects in women [26]. In addition, a different response to statin therapy has been observed in female patients, mainly due to hormonal imbalance [27,28,29].

### 4.2. Angiographic and Biohumoral Data Associated with the Four Determinants of Residual Risk and Clinical Outcomes

Our study showed that compared to STEMI, NSTEMI patients had a significantly higher number of total coronary stenoses, culprit stenoses, and non-culprit stenoses, suggesting that coronary atherosclerosis has a greater extent in NSTEMI patients. In addition, non-culprit lesion percentage stenosis was significantly greater in NSTEMI patients as compared to STEMI patients, suggesting a greater plaque burden.

The combination of a greater extent of coronary atherosclerosis and a higher plaque burden may explain the higher risk of both MACE and non-fatal RCE seen in NSTEMI patients.

A previous paper, conducted using computed tomography angiography in a large general population, showed that the risk of death or myocardial infarction was highest in patients with extensive coronary disease [19]. The PROSPECT study used intravascular ultrasound to identify predictors of recurrent events in coronary artery disease patients. It found that a small luminal area and a higher plaque burden had the most significant impact on the risk of recurrence [30].

In contrast, neither DM nor CKD had a different number of coronary stenoses or a greater degree of non-culprit lesion percentage stenosis. A similar result was also observed between female and male patients.

Interestingly, DM patients had a higher frequency of bifurcation involvement, and they also exhibited a trend toward a higher risk of non-fatal RCE.

A previous optical coherence tomography (OCT) study showed that DM patients presenting with ACSs had a higher frequency of lipid plaque with a thin fibrous cap in both culprit and non-culprit sites as compared to non-DM patients [31].

The higher prevalence of plaque vulnerability features may account for the increased risk observed in DM patients.

In our study, CKD patients had a higher frequency of LM involvement. Previous studies showed a higher frequency of necrotic core and dense calcium in the non-culprit plaque of CKD patients [32,33], suggesting a more stable plaque phenotype. We showed that CKD patients were associated with an increased risk of MACE driven by a higher rate of death from any cause and that higher creatinine values at admission were an independent predictor of MACE. Of note, non-fatal RCEs were not significantly different as compared to non-CKD patients, probably due to the lower vulnerability of non-culprit plaques in CKD patients.

Finally, female patients showed smaller culprit coronary vessels as compared to male subjects. This confirms the findings of a previous OCT study, which showed that female patients have smaller vessel dimensions [34]. In addition, female patients showed greater signs of plaque vulnerability with advanced age, probably due to the lack of the antiatherosclerotic effect of estrogen after menopause [35].

In our study, female patients were older than male subjects. However, the high rate of potent P2Y12 inhibitors and high-intensity lipid-lowering drugs may have prevented subsequent events in female patients.

Finally, laboratory data analysis showed that the four determinants of residual risk were all associated with lower hemoglobin levels at hospital admission. Additionally, a lower hemoglobin level at admission was an independent predictor of MACE. Our data is in line with a previous work that showed the impact of admission anemia on the excess risk of all-cause mortality and myocardial infarction at 6 months after ACSs [36]. Anemia was detected in up to 24% of ACS patients and was associated not only with early but also with late all-cause mortality [37]. The reasons that connect anemia to the worse outcome are not only related to the impaired local oxygen release and subsequent ischemia but also to the reduced regulation of nitric oxide-mediated endothelial function due to a lower number of erythrocytes [38]. In fact, erythrocytes have a central role in endothelial and microvascular function [39]. Other putative reasons can be considered a potential undertreatment of anemic patients with antithrombotic treatments or the greater comorbidity prevalence (i.e., DM, CKD, heart failure, and history of bleeding) seen in patients with anemia [37].

## 5. Conclusions

In conclusion, we found that being female sex, having NSTEMI as a type of ACS, having DM, and having CKD were all associated with MACE at a 5-year follow-up time. Female sex was not an independent predictor of MACE after adjusting for other comorbidities. NSTEMI patients showed an increased risk of subsequent non-fatal RCE; a trend was also observed for DM patients. A greater extent of coronary atherosclerosis was seen in NSTEMI patients, while DM and CKD patients more frequently involved bifurcation and LM lesions, respectively.

Lower hemoglobin values were common among the four determinants of residual risk and represented an independent predictor of MACE.

## 6. Limitations

The present work has some limitations.

Firstly, we evaluated death from any cause without differentiating between cardiac and other causes. Further analysis of the causes of death was not feasible due to the retrospective nature of the current investigation.

Secondly, the lack of intravascular imaging information about plaque composition was another limitation. Nevertheless, earlier studies have assessed the features that increase the likelihood that plaques would progress in the future [40,41,42].

Furthermore, we included a large cohort of ACS patients who may have precluded performing an intravascular imaging examination on a regular basis.

Thirdly, as this is a retrospective study, it was not possible to collect sufficient data related to left ventricle ejection fraction. However, we included data on peak high-sensitivity troponin I, which can serve as a surrogate marker of acute left ventricle injury.

## Figures and Tables

**Table 1:** Baseline clinical features.

Male Sex, n (%)	1056 (75.2)
Age, years	68.6 (12.3)
STEMI, n (%)	816 (58.1)
NSTEMI, n (%)	589 (41.9)
CKD, n (%)	446 (31.7)
DM, n (%)	344 (24.5)
Dyslipidemia, n (%)	677 (48.2)
Smoking status	
Never, n (%)	866 (61.6)
Present, n (%)	296 (21.1)
Past, n (%)	243 (17.3)
BMI, Kg/m^2^	27.3 (4.4)
Weight, Kg	78.1 (15.3)
Height, cm	168.8 (8.7)
Treatment	
Aspirin, n (%)	1177 (83.8)
Clopidogrel, n (%)	565 (40.2)
Prasugrel, n (%)	424 (30.2)
Ticagrelor, n (%)	348 (24.8)
High-intensity statin, n (%)	1311 (93.3)
Ezetimibe, n (%)	373 (26.5)
Insulin, n (%)	123 (8.8)
OAD drug, n (%)	200 (14.2)

Data is expressed as mean (standard deviation) or number of cases (percentage) as appropriate. BMI stands for body mass index. OAD stands for oral antidiabetic.

**Table 2:** Coronary angiography analysis.

Total N° of Coronary Stenoses	3.2 (1.6)
N° of Culprit stenoses	1.5 (0.7)
Culprit lesion stenosis	93.2 (8.6)
Culprit RVD, mm	3.2 (0.5)
Culprit MLD, mm	0.26 (0.26)
Culprit stenosis length, mm	27.6 (14.8)
Culprit vessel	
LM, n (%)	29 (2.1)
LAD, n (%)	637 (45.3)
LCx, n (%)	303 (21.5)
RCA, n (%)	436 (31)
Bifurcation, n (%)	406 (28.9%)
N° of deployed stent	1.3 (0.7)
Stent diameter, mm	3.2 (0.4)
Non-culprit lesion stenosis, %	51.6 (18.0)
N° of non-culprit stenosis	1.9 (1.5)

Data is expressed as mean (standard deviation) or number of cases (percentage), as appropriate. N° stands for number, LM for left main, LAD for left anterior descending, LCx for left circumflex, and RCA for right coronary artery.

**Table 3:** Laboratory data at hospital admission for the entire studied population.

LDL-Cholesterol, (mg/dL)	124.4 (36.8)
HDL-cholesterol, (mg/dL)	43.4 (15.8)
Triglycerides, (mg/dL)	132.5 (76.2)
Creatinine, (mg/dL)	1.2 (4.0)
Glycemia, (mg/dL)	135.8 (58.7)
HbA1c, %	7.1 (1.6)
Platelets, (1000/µL)	227.6 (71.4)
Hemoglobin, (g/dL)	13.8 (1.9)
White blood cells, 1000/mm^3^	10.1 (4.1)
C-reactive protein, (mg/dL)	3.3 (5.5)
Fibrinogen, (mg/dL)	476.4 (163.5)
Uric acid, (mg/dL)	6.1 (1.7)
Troponin I, (ng/L)	2422.3 (2773)

Data is expressed as mean (standard deviation).

**Table 4:** Laboratory data at 1-month and 12-month follow-up times for the entire studied population.

Parameter	At 1 Month	At 12 Months
LDL-cholesterol, (mg/dL)	79.2 (27.6)	75.9 (25.1)
HDL-cholesterol, (mg/dL)	44.0 (11.5)	44.5 (10.9)
Creatinine, (mg/dL)	1.1 (0.8)	1.2 (2.2)
Glycemia, (mg/dL)	111.8 (35.1)	114.6 (36.5)
HBA1c, %	6.6 (1.2)	6.6 (1.1)
Platelets, (1000/µL)	225.5 (68.9)	222.3 (72.4)
Triglycerides, (mg/dL)	127.5 (73.2)	124.2 (61.8)
Hemoglobin, (mg/dL)	13.7 (1.7)	13.7 (1.7)

Data is expressed as mean (standard deviation).

**Table 5:** Main and secondary clinical endpoints.

	Entire Cohort (1405)	Female (349)	Male (1056)	*p*	STEMI (816)	NSTEMI (589)	*p*	DM (344)	Non-DM (1061)	*p*	CKD (446)	Non-CKD (959)	*p*
MACE, n (%)	485 (34.5)	150 (43)	335 (31.7)	0.0001	229 (28.1)	256 (43.5)	0.0001	140 (40.7)	345 (32.5)	0.006	210 (47.1)	275 (28.7)	0.0001
Death from any cause, n (%)	372 (26.5)	119 (34.1)	253 (24)	0.001	188 (23)	184 (31.2)	0.001	105 (28.2)	267 (25.2)	0.057	185 (41.5)	187 (19.5)	0.0001
RCE, n (%)	152 (10.8)	41 (11.7)	111 (10.5)	0.551	50 (6.1)	102 (17.3)	0.0001	46 (13.4)	106 (10.0)	0.089	49 (11)	103 (10.7)	0.927

MACE (major adverse cardiovascular events) included death from all causes and non-fatal RCE; RCE (recurrent coronary events) only included non-fatal recurrent coronary events.

**Table 6:** Univariate and multivariate logistic regression for MACE as dependent variable.

	Univariate Logistic Regression				Multivariate Logistic Regression			
	Beta	*p* Value	OR	95% CI	Beta	*p* Value	OR	95% CI
Female gender	−0.48	0.0001	0.61	0.48–0.79	0.32	0.894	1.38	0.62–3.07
NSTEMI presentation	0.67	0.0001	1.97	1.57–2.46	0.91	0.0001	2.48	1.29–4.79
Age	0.07	0.0001	1.07	1.06–1.08	0.01	0.221	1.01	0.97–1.05
CKD	0.79	0.0001	2.21	1.75–2.79	0.14	0.769	1.19	0.799–1.98
Total N° of stenoses	0.10	0.001	1.11	1.04–1.19	0.09	0.848	1.09	0.74–1.62
N° of culprit stenoses	0.15	0.029	1.17	1.01–1.34	0.21	0.646	1.23	0.74–2.07
Stent diameter	−0.24	0.047	0.78	0.61–0.99	0.00	0.935	1.00	0.63–1.58
NC lesion stenosis	0.01	0.018	1.008	1.001–1.015	0.00	0.844	1.00	0.99–1.02
Diabetes mellitus	0.35	0.006	1.42	1.10–1.83	0.89	0.0001	2.44	1.66–3.56
N° of NC stenoses	0.10	0.004	1.11	1.03–1.19	0.05	0.797	1.05	0.72–1.53
Hb at admission	−0.34	0.0001	0.70	0.66–0.75	−0.116	0.020	0.89	0.73–0.97
Weight	−0.1	0.0001	0.98	0.97–0.99	0.001	0.965	1.00	0.99–1.02
Height	−0.4	0.0001	0.95	0.94–0.96	0.001	0.889	1.00	0.97–1.04
Creatinine at admission	0.01	0.362	1.01	0.98–1.05	0.43	0.028	1.54	1.09–2.17
Glycemia at admission	0.003	0.016	1.003	1.001–1.005	0.001	0.685	1.00	1.0–1.01
LDL-cholesterol at admission	−0.01	0.0001	0.99	0.98–0.99	−0.001	0.681	1.00	0.99–1.00
HDL-cholesterol at admision	−0.01	0.024	0.98	0.97–0.99	−0.002	0.898	0.99	0.97–1.01
High-sensitivity troponin I	0.001	0.013	1.000	1.00–1.00	0.001	0.933	1.00	1.00–1.00

The model as a whole explained between 15.9% (Cox and Snell R square) and 21.9% (Nagelkerke R square) of the variance in recurrent coronary events and correctly classified 75.7% of cases. N° stands for number, and Hb stands for hemoglobin. MACE (major adverse cardiovascular events) included death from all causes and non-fatal recurrent coronary events.

## Data Availability

The original contributions presented in this study are included in the article. Further inquiries can be directed to the corresponding author.

## Abbreviations

The following abbreviations are used in this manuscript:

ACS	acute coronary syndrome
ANOVA	analysis of variance
CKD	chronic kidney disease
DM	diabetes mellitus
DS	diameter stenosis
eGFR	estimated glomerular filtration rate
KDIGO	kidney disease improving global outcomes
LM	left main
MACE	major adverse cardiovascular events
MDRD	modification of diet in renal disease
MLD	minimum lumen diameter
NSTEMI	non-ST-segment-elevation myocardial infarction
OCT	optical coherence tomography
PCI	percutaneous coronary intervention
RCE	recurrent coronary event
RVD	reference vessel diameter
SAGER	Sex and Gender Equity in Research
STEMI	ST-segment–elevation myocardial infarction
